# Complete Genome Analysis of Canine Respiratory Coronavirus

**DOI:** 10.1128/genomeA.00093-12

**Published:** 2013-01-31

**Authors:** Seong-in Lim, Sarah Choi, Ji-Ae Lim, Hye-Young Jeoung, Jae-Young Song, R. C. dela Pena, Dong-Jun An

**Affiliations:** Animal, Plant, and Fisheries Quarantine and Inspection Agency, Anyang, Gyeonggi, Republic of Korea

## Abstract

The canine respiratory coronavirus (CRCoV) K37 strain of the family *Coronaviridae*, group 2, was isolated in South Korea. Its genome was analyzed by nucleotide sequencing and was determined to have 31,029 bp. The small open reading frames situated between the spike and envelope genes of most of the CRCoV strains (except the CRCoV 4180 strain) were found to encode three nonstructural proteins (4.9 kDa, 2.7 kDa, and 12.8 kDa), while those of bovine coronavirus (BCoV) encode another three nonstructural proteins (4.9 kDa, 4.8 kDa, and 12.7 kDa) and those of a recently isolated bovine respiratory coronavirus (BRCoV) were found to encode only two nonstructural proteins (4.9 kDa and 12.7 kDa). The differences in the genes encoding these small nonstructural proteins may be associated with the emergence of highly similar viruses in different hosts.

## GENOME ANNOUNCEMENT

Canine respiratory coronavirus (CRCoV) belongs to coronavirus group 2 and is a causative agent of canine infectious respiratory disease (CIRD). This virus emerged in England as a novel pathogen in the respiratory tracts of dogs suffering from severe respiratory disease (1). The genomic organization of the CRCoV 4182 prototype strain isolated from a dog in England reveals that its major structural and nonstructural proteins are genetically related to bovine coronavirus (BCoV) proteins but that the genes encoding two or three small nonstructural proteins situated between the spike (S) and envelope (E) proteins differ from those of BCoV (2, 3). To date, there are no complete genomic sequence data available publicly for CRCoV; hence, in this study, we aimed to analyze the full genomic sequence of the K37 strain that was derived from a Korean dog with clinical respiratory symptoms (2).

Total RNA for CRCoV K37 was extracted using the microcolumn-technique-based QIAamp viral RNA minikit (Qiagen), and cDNA was amplified using a one-step reverse transcriptase (RT)-PCR kit (Qiagen). Specific primers for amplification of the genome were designed based on the genome sequences of BCoV. RT-PCR amplification products were cloned into the pGEM-T plasmid and were sequenced with T7 and SP6 sequencing primers using an Applied Biosystems (ABI) Prism 3730xi DNA sequencer.

Comparative analyses between open reading frame 1ab (ORF1ab), ORF1a, 32-kDa nonstructural protein (32-kDa NSP), hemagglutinin-esterase (HE), spike (S), 12.7-kDa (or 12.8-kDa) NSP, envelope (E), membrane (M), nucleocapsid (N), and internal N protein (I) gene sequences of CRCoV and bovine respiratory coronavirus (BRCoV) and comparison with the sequences of the reference BCoV strain showed that CRCoV and BRCoV shared high homology to the reference BCoV strain: 99.5% (CRCoV) and 99.8% (BRCoV) for ORF1ab, 99.0% and 99.8% for ORF1a, 97.6% and 99.4% for the 32-kDa NSP, 98.1% and 99.5% for HE, 96.4% and 98.8% for the spike protein, 98.2% and 100% for the 12.7-kDa (12.8-kDa) NSP, 99.6% and 100% for the envelope protein, 98.9% and 99.6% for the membrane protein, 97.9% and 99.5% for the nucleocapsid protein, and 97.8% and 99.7% for the I protein. The transcription regulatory sequences (TRSs) of CRCoV K37 occur in two forms, the CUAAAC type (upstream of the genes encoding the 32-kDa NSP, HE, S, the 12.8-kDa NSP, N, and I) and the CCAAAC type (upstream of the genes encoding the 4.9-kDa and 2.7-kDa NSPs, E, and M). The HE protein of the K37 strain was found to contain nine potential glycosylation sites upon analysis with the NetNGlyc 1.0 server, while CRCoV strain 240-05 was found to have eight potential N-glycosylation sites (4).

In summary, phylogenetic analyses of the genome sequences of CRCoV K37 and the reference coronaviruses obtained from GenBank revealed genetic lineages through Mega 4.1 program analyses (5). CRCoV belongs to the 2a subgroup, with HCoV, BCoV, BRCoV, mouse hepatitis virus (MHV), pigeon herpes encephalomyelitis virus (PHEV), and coronavirus HKU1 (CoV-HKU1) of coronavirus group 2.

### Nucleotide sequence accession number.

The complete genome sequence of the CRCoV K37 strain was deposited at GenBank under the accession no. JX860640.
